# Serial dependence in the perceptual judgments of radiologists

**DOI:** 10.1186/s41235-021-00331-z

**Published:** 2021-10-14

**Authors:** Mauro Manassi, Cristina Ghirardo, Teresa Canas-Bajo, Zhihang Ren, William Prinzmetal, David Whitney

**Affiliations:** 1 School of Psychology, King’s College, University of Aberdeen, Aberdeen, UK; 2 Department of Psychology, University of California, Berkeley, CA USA; 3 Helen Wills Neuroscience Institute, University of California, Berkeley, CA USA; 4 Vision Science Group, University of California, Berkeley, CA USA

**Keywords:** Serial dependence, Visual search, Radiological screening, Priming, Sequential effects, Sequential dependence

## Abstract

In radiological screening, clinicians scan myriads of radiographs with the intent of recognizing and differentiating lesions. Even though they are trained experts, radiologists’ human search engines are not perfect: average daily error rates are estimated around 3–5%. A main underlying assumption in radiological screening is that visual search on a current radiograph occurs independently of previously seen radiographs. However, recent studies have shown that human perception is biased by previously seen stimuli; the bias in our visual system to misperceive current stimuli towards previous stimuli is called serial dependence. Here, we tested whether serial dependence impacts radiologists’ recognition of simulated lesions embedded in actual radiographs. We found that serial dependence affected radiologists’ recognition of simulated lesions; perception on an average trial was pulled 13% toward the 1-back stimulus. Simulated lesions were perceived as biased towards the those seen in the previous 1 or 2 radiographs. Similar results were found when testing lesion recognition in a group of untrained observers. Taken together, these results suggest that perceptual judgements of radiologists are affected by previous visual experience, and thus some of the diagnostic errors exhibited by radiologists may be caused by serial dependence from previously seen radiographs.

## Significance statement

In a medical screening setting, radiologists repeatedly search for signs of tumors in radiological scan images, classifying them, judging their size, class, position and so on. An underlying assumption about visual search in this setting is that current perceptual experience is independent of our previous perceptual experience. Here, we show that perceptual judgments of radiologists are biased by serial dependence. We found that radiologists’ recognition of simulated lesions was strongly biased by their past visual experience. This source of error, unlike a mere response bias, extended over 10 seconds back in time (was temporally tuned), occurred only between similar lesions (was featurally tuned), and within a limited spatial region (was spatially tuned). Our experiments provide evidence for a newly pinpointed source of error in radiological screening. Crucially, our results show limited and precise boundaries within which the detrimental effects of serial dependence occur in radiologists, and open the path to potential strategies which may mitigate their detrimental effects.

## Introduction

Cancer diagnosis in medical images is crucial for the health of millions of people, but it is still far from perfect. For example, within mammography, false negative and false positive rates have been reported to be 0.15% and 9%, respectively (Nelson et al., 2016). Some of these misdiagnoses are due to misperceptions and misinterpretations of radiographs by clinicians (Berlin, 2007; Croskerry, 2003). Interpretive errors in radiology are defined as the discrepancy in interpretation between the radiologist and peer consensus (Bruno et al., 2015; Waite et al., 2017), and it has been proposed that perceptual errors account for 60–80% of the total amount (Funaki et al., 1997; Kim & Mansfield, 2014).

Some sources of interpretive error have been identified and characterized, including search and recognition errors (Carmody et al., 1980; Nodine et al., 1996), cognitive biases (Croskerry, 2003; Lee et al., 2013), search satisfaction (Ashman et al., 2000; Berbaum & Franken Jr, 2011), subsequent search misses (Birdwell et al., 2001; Boyer et al., 2004; Harvey et al., 1993), and low prevalence (Wolfe et al., 2005, 2007; Rich et al., 2008; Menneer et al., 2010; Evans et al., 2013; Horowitz, 2017; Kunar et al., 2017). However, some other errors in cancer image interpretation are still without explanation (Bruno et al., 2015; Waite et al., 2017, 2019). Given the importance of this issue, a great deal of research has been carried out in the last decades to understand how to identify and characterize the source of these mistakes in order to mitigate them as much as possible.

When looking at a radiograph, clinicians are typically asked to localize lesions (if present), and then to classify them by judging their size, class, and so on. Importantly, during this visual search task, radiologists often examine dozens or hundreds of images in batches, sometimes seeing several related images one after the other. During this process, a main underlying assumption is that radiologists’ percepts and decisions about a current image are completely independent of prior perceptual events. Recent theoretical and empirical research has raised the possibility that this is not true.

The visual system is characterized by visual serial dependency, a type of sequential effect in which what was previously seen influences (captures) what is seen and reported at this moment (Cicchini et al., 2014; Fischer & Whitney, 2014). Serial dependencies can manifest in several domains, such as perception (Cicchini et al., 2017, 2018; Fischer & Whitney, 2014; Manassi et al., 2018), decision making (Abrahamyan et al., 2016; Fernberger, 1920), and memory (Barbosa & Compte, 2020; Fornaciai & Park, 2020; Kiyonaga et al., 2017), and they occur with a variety of features and objects, including orientation, position, faces, attractiveness, ambiguous objects, ensemble coding of orientation, and numerosity (Bliss et al., 2017; Corbett et al., 2011; Fischer & Whitney, 2014; Fornaciai & Park, 2018; Kondo et al., 2012; Liberman et al., 2018; Manassi et al., 2017; Taubert & Alais, 2016; Taubert et al., 2016a; Wexler et al., 2015; Xia et al., 2016). Serial dependence is characterized by three main kinds of tuning. First, feature tuning: serial dependence occurs only between similar features and not between dissimilar ones (Fischer & Whitney, 2014; Fritsche et al., 2017; Manassi et al., 2017, 2018). Second, temporal tuning: serial dependence gradually decays over time (Fischer & Whitney, 2014; Manassi et al., 2018; Wexler et al., 2015). Third, spatial tuning: serial dependence occurs only within a limited spatial window; it is strongest when previous and current objects are presented at the same location, and it gradually decays as the relative distance increases (Bliss et al., 2017; Collins, 2019; Fischer & Whitney, 2014; Manassi et al., 2018). In addition, attention is a necessary component for serial dependence (Fischer & Whitney, 2014; Fritsche & de Lange, 2019; Kim et al., 2020).

The empirical results above prompted our theoretical suggestion that perception occurs through Continuity Fields—temporally and spatially tuned operators or filters that bias our percepts towards previous stimuli through serial dependence (Alais et al., 2017; Cicchini et al., 2017; Fischer & Whitney, 2014; Taubert et al., 2016a, 2016b). Continuity Fields are a helpful, beneficial mechanism for promoting perceptual stability because they produce a smoothed percept that better matches the autocorrelations in the world in which we live (Fischer & Whitney, 2014; Liberman et al., 2014; Manassi et al., 2017). In contrast to the highly structured and stable physical world, retinal images are constantly changing due to external and internal sources of noise and discontinuities from eye blinks, occlusions, shadows, camouflage, retinal motion, and other factors. Rather than processing each momentary image or object as being independent of preceding ones, the visual system favors recycling previously perceived features and objects. By incorporating serially dependent perceptual interpretations, the visual system smooths perception (and decision making and memory; Kiyonaga et al., 2017) over time and helps us perceive a continuous and stable world despite noise and change.

The benefits of serial dependence arise because the world we encounter is *usually* autocorrelated. But it is not always. In some artificial, human-contrived, situations the world is not autocorrelated. One obvious example of this are visual stimuli attended in laboratory experiments (in visual psychophysics, cognition, psychology, neurophysiology, and many other domains). Often stimuli are randomly ordered, with the assumption that trials are treated independently by the brain (Mulder et al., 2012; Winkel et al., 2014). Serial dependence negatively impacts the ability to measure performance in these cases (Fischer & Whitney, 2014; Fründ et al., 2014; Liberman et al., 2014).

Visual search in clinical settings, such as reading radiographs or pathology slides, is an even more striking example where stimuli may not be autocorrelated. When seeing and judging lesions under such circumstances, serial dependence could introduce a bias in perceptual judgments that may result in a significant reduction in sensitivity and increase in errors. The negative impacts of serial dependence in search tasks would be especially prominent in cases where there is low signal, high noise, high uncertainty, or where fine discriminations are required (Bliss et al., 2017; Cicchini et al., 2014, 2017, 2018; Fischer & Whitney, 2014; Manassi et al., 2017). These are exactly the challenging situations that radiologists routinely face when searching scans. We hypothesize that because of serial dependence, radiologists’ perceptual decisions on any given current radiograph could be biased towards the previous images they have seen. To preview our results, we measured recognition of simulated tumors in trained clinicians and found that their perceptual judgments were significantly affected by serial dependence.

## Method

### Observers and apparatus

All experimental procedures were approved by and conducted in accordance with the guidelines and regulations of the UC Berkeley Institutional Review Board. Participants provided informed consent in accordance with the IRB guidelines of the University of California at Berkeley. All participants had normal or corrected-to-normal vision, and were all naïve to the purpose of the experiment. Fifteen trained radiologists (gender: 4 female, 11 males; qualification: 11 experts, 3 residents, & 1 fellow; age: 27–72 years) participated in Experiment 1. They were recruited at RSNA, Radiological Society of North America Annual Meeting (Chicago, US December 1st–6th, 2019). Of the fifteen, two participants did not complete the study, and their data were excluded. Eleven non-expert observers (7 female; aged 19–21 years) participated in Experiment 2. Sample size was determined based on radiologists’ availability at RSNA, and was similar to current studies of serial dependence (Cicchini et al., 2018; Manassi et al., 2019; Pascucci et al., 2017). Eleven non-expert observers (7 female; aged 19–21 years) participated in Experiment 2. They were recruited from a student pool at UC Berkeley.

Stimuli were generated on a 13.3 inch 2017 MacBook Pro with a 28.7 cm × 18 cm screen with PsychoPy (Peirce, 2007, 2009). The refresh rate of the display was 60 Hz and the resolution 1440 × 900 pixels. Stimuli were viewed from a distance of approximately 57 cm. Observers used a laptop keyboard for all responses.

### Stimuli and design

**Fig. 1:**
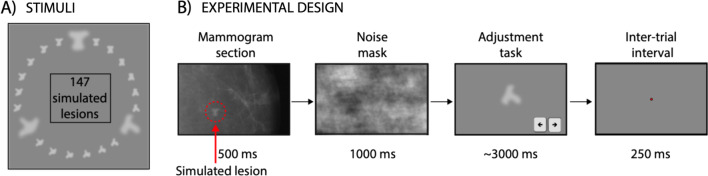
Stimuli and design of the Experiments 1 and 2. **A** We created three objects with random shapes (prototypes *A*/*B*/*C*, shown in a bigger size) and generated 48 morph shapes in between each pair (147 shapes in total). We used these shapes as simulated lesions during radiological screening. **B** Observers were presented with a random shape (simulated lesion) hidden in a mammogram section, followed by a noise mask. Radiologists were then asked to adjust the shape to match the simulated lesion they previously saw, and pressed spacebar to confirm. During the inter-trial-interval, a red fixation dot appeared in the center. The size of the shape adjustment is identical to the size of the simulated lesion, but it was enlarged for illustrative purposes. After a 250 ms inter-trial interval, the next trial started

To simulate the screening performed by radiologists, we created three objects with random shapes and generated 48 morph shapes in between each pair (147 shapes in total; Fig. 1A). We used these shapes as simulated lesions. On each trial, radiologists viewed a random simulated lesion superimposed on a mammogram section and were then asked to adjust a shape to match the simulated lesion they previously saw. The stimuli consisted of light-gray shapes based on 3 original prototype shapes (A/B/C; Fig. 1A). A set of 48 shape morph shapes was created between these prototypes, resulting in a morph continuum of 147 shapes. The shapes were approximately 3.7° width and height. Each shape was blurred by using a gaussian blur function in OpenCV with a gaussian kernel size of 1.55°. On each trial, a random shape was presented at a random angular location relative to central fixation (0.35°) in the peripheral visual field (4.4° eccentricity, from center to center). The shape was embedded in a random mammogram (30% transparency level) and was presented for 500 ms (Fig. 1B). Mammograms were taken from The Digital Database for Screening Mammography (Bowyer et al., 1996; 100 possible alternatives) and enlarged to fit the screen. The mammograms (~ 2000 × 4500 pixels) were enlarged three times and cut at a central position such that about 15% of each x-ray was displayed. This resulted in breast tissue covering the entire screen. Next, we presented a mask composed of random Brownian noise background (1/f^2^ spatial noise). After the mask, a random shape drawn from the morph continuum (width and height: 3.7°; color: light-gray) appeared at the fixation point location, and observers were asked to adjust the shape to match the perceived shape using the left/right arrow keys (continuous report, adjustment task; left–right arrow keys to adjust the shape). The starting shape was randomized on each trial. Observers were allowed to take as much time as necessary to respond and pressed the spacebar to confirm the chosen shape. Following the response and a 250 ms delay, the next trial started.

During the experiment, observers were asked to continuously fixate a red dot in the center (0.35° radius). On each trial, they were first presented with a shape in a random location at 4.4° eccentricity, followed by a noise mask (Fig. 1). Observers were then asked to adjust a shape to match the one they previously saw (adjustment task). Observers performed 3 blocks of 85 trials each (Fig. 1B). In a preliminary session, observers completed a practice block of 10 trials. Mean adjustment time was 3240 ± 804 ms in Experiment 1 and 2980 ± 578 ms in Experiment 2. The only difference between Experiment 1 and 2 were the participants. In Experiment 1, we tested trained radiologists, whereas in Experiment 2, we tested students from the UC Berkeley population. Equipment and experimental design were otherwise identical.

## Data analysis

### Feature tuning analysis

We measured response errors on the adjustment task to determine whether a subject’s judgment of each simulated lesion was influenced by the previously seen lesions. Response error was computed as the shortest distance along the morph wheel between the match morph and the target one (current response – current shape morph). For each participant’s data, trials were considered lapses and were excluded if adjustment error exceeded 3 standard deviations from the absolute mean adjustment error or if the response time was longer than 20 s. Less than 2% of data was excluded on average.

Response error was compared to the difference in shape between the current and previous trial, computed as the shortest distance along the morph wheel between the previous target lesion (n-back) and the current target shape (current response – current shape morph). We quantified feature tuning by fitting a von Mises distribution to each subject’s data points (see details below). Additionally, for each observer, we computed the running circular average within a 20 morph units window. Figure 3A-B shows the average of the moving averages across all the observers, and the corresponding von Mises fit. Figure 3E-F shows the half-amplitudes von Mises distribution for individual observers.

### Temporal tuning analysis

We quantified temporal tuning by fitting a derivative of von Mises to each subject’s data using the following equation:

$$y = - \frac{{a\kappa \sin \left( {x - \mu } \right)e^{{\kappa \cos \left( {x - \mu } \right)}} }}{{2\pi I_{0} \left( \kappa \right)}}$$

where parameter $y$ is response error on each trial, $x$ is the relative orientation of the previous trial, $a$ is the amplitude modulation parameter of the derivative-of-von-Mises, $\mu$ indicates the symmetry axis of the von Mises derivative, $\kappa$ indicates the concentration of the von Mises derivative, and $I_{0} \left( \kappa \right)$ is the modified Bessel function of order 0. In our experiments, $\mu$ is set to 0. We fitted the von Mises derivative using constrained nonlinear minimization of the residual sum of squares. As a measure of serial dependence, we reported half the peak-to-trough amplitude of the derivative-of-von-Mises (Figure 3E, F). We used the half amplitude of the von Mises, the $a$ parameter in the above equation, to measure the degree to which observers’ reports of simulated lesions were pulled in the direction of n-back simulated lesions. For example, if subjects’ perception of a lesion was repelled by the 1-back simulated tumor (e.g., because of a negative aftereffect), or not influenced by the 1-back lesion (because of independent, bias-free perception on each trial), then the half-amplitude of the von Mises should be negative or close to zero, respectively.

For each subject’s data, we generated confidence intervals by calculating a bootstrapped distribution of the model-fitting parameter values. For each observer, we resampled the data with replacement 5000 times (Efron & Tibshirani, 1986). The relationship on each trial between response error and relative difference in shape (between the current and previous trial) was maintained. On each iteration, we fitted a new von Mises to obtain a bootstrapped half-amplitude and width for each subject.

Previous research recently showed that individual observers can have idiosyncratic biases in object recognition and localization, which are unrelated to serial dependence. For example, there are individual stable differences in perceived position and size, originating from a heterogeneous spatial resolution that carries across the visual hierarchy (Kosovicheva & Whitney, 2017; Wang et al., 2020). For this reason, we conducted an additional control analysis to remove such potential unrelated biases before fitting the von Mises derivative function. We plotted observer’s error values (current response – current shape morph) as a function of the actual stimulus presented (current shape morph), and fit a radial basis function (30 Gaussian Kernels used) to the data. This allowed us to quantify the idiosyncratic bias for each observer. For example, observers may make a consistent error in reporting a simulated lesion of 20 morph units as being 10, thus creating a systematic error of − 10 morph units. Conversely, if there was no systematic error, all error would approximate zero. We then regressed out the bias quantified by the radial basis fit by subtracting it from the observer’s error. This subtraction left us with residual errors that did not include the idiosyncratic biases unrelated to serial dependence. Importantly, the addition of this control analysis—removing systematic biases unrelated to serial effects—had no significant impact on the serial dependence results. It did not generate or increase the measured serial dependence.

As an additional method to rule out potential unrelated biases on the serial dependence effect, we explored the effect of future trials on the current response (Fornaciai & Park, 2020; Maus et al., 2013). That is, we compared the current trial response error to the difference in shape between the current and following trial (n-forward). Since observers have not seen the future trial shape, their current response in a given trial should not be in any ways related to the shape that will be presented to them next.

### Spatial tuning analysis

In order to measure the spatial tuning of serial dependence, we binned trials according to the distance between the current and previous shape angular locations (Fig. 4). First, we divided trials from each observer into 3 main relative angular distance groups: 0°–60°, 61°–120°, and 121°–180° for 1-back trials. For example, a relative angular distance of 0° indicates that previous and current lesions were presented at the same location (for example, 45° and 45° of angular distance in previous and current trials). Similarly, a relative angular distance of 60° indicates that previous and current lesions were presented at 30° and 90° of angular distance. The distance between successive shape locations was computed as $\sqrt {\left( {x{\text{current}} - x{\text{previous}}} \right)^{2} + \left( {y{\text{current}} - y{\text{current}}} \right)^{2} .}$ Second, we extracted 60 random trials from each observer for each distance group, and collapsed all the trials from all the observers in three super-subject groups. Third, for each super-subject we fitted a derivative of von Mises and computed the half amplitudes. Fourth, we performed a regression line analysis across the three half amplitudes of the distance groups. For each super-subject, this analysis yielded a slope of the regression line, which reflects how much serial dependence varies as a function of distance between sequential stimuli. We repeated the procedure 5000 times, by resampling the data with replacement on each iteration.

## Results

We tested whether serial dependence influenced recognition of simulated lesions when viewing consecutive images of mammogram tissues in radiologists and untrained observers. Response error (*y*-axis) was computed as the shortest distance along the morph wheel between the match shape and the simulated lesion. Average response error was similar across groups; 9.2 ± 1.8 morph units in Experiment 1 (radiologists) and 8.9 ± 1.8 in Experiment 2 (untrained observers; *t*(22) = 0.34, *p* = 0.74).

**Fig. 2:**
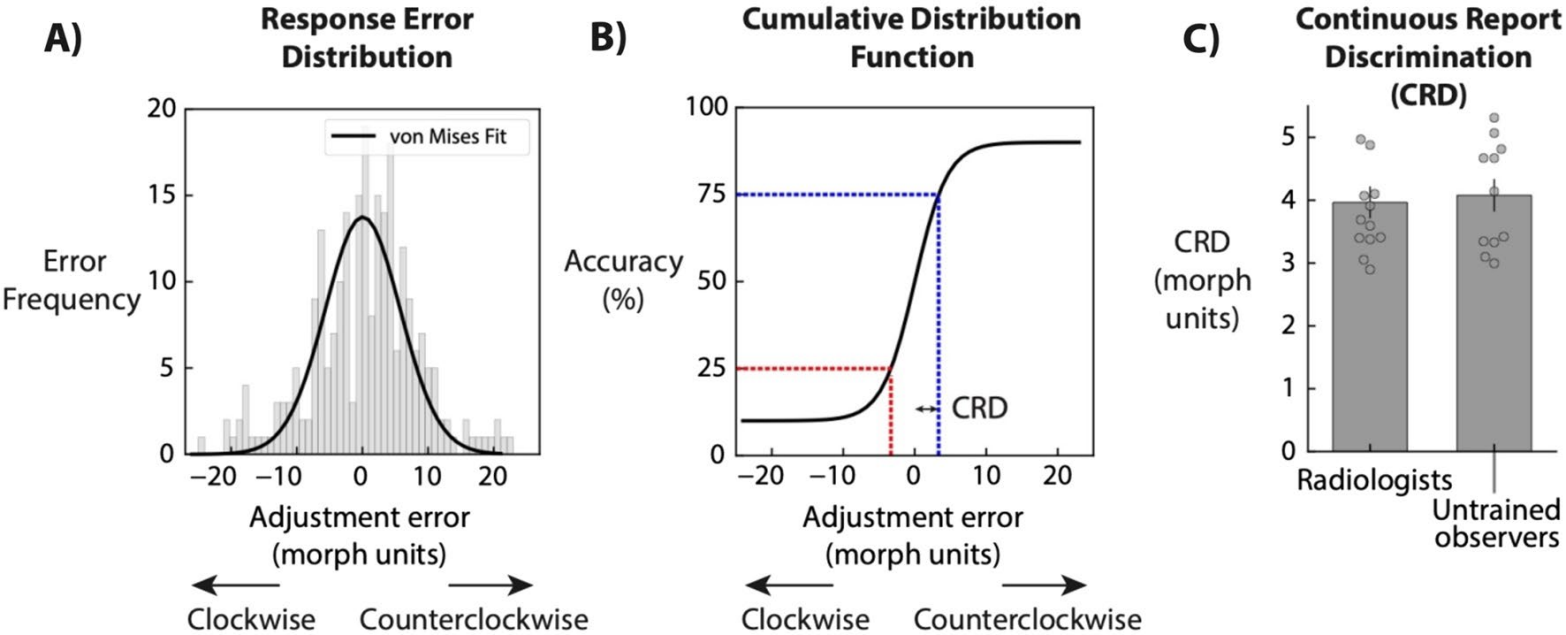
Continuous Report Discrimination index (C.R.D). **A** For each observer, we plotted a frequency histogram of the adjustment errors and fitted a Von Mises to quantify adjustment performance. **B** We then converted the von Mises fit into a Cumulative Distribution Function. Continuous Report Discrimination index was calculated by taking the half difference between 25 and 75th percentile in terms of adjustment error morph units. **C** Each dot shows CRD index for individual observers in the two groups. Bars indicate average in Experiment 1 and 2, and error bars indicate standard error

To further quantify discriminability of the simulated lesions, we fit a von Mises function to each observer's response error frequency distribution (Fig. 2A) and computed the corresponding Cumulative Distribution Function (CDF; Fig. 2B). The CDF was generated with a ceiling and floor parameters of 0.1 and 0.9, respectively, and a free *x*-axis shift parameter to allow for any observers’ bias to be taken into account. For each observer's individual CDF, a Continuous Report Discrimination index (C.R.D.) was defined as half of the difference between the 25th and 75th percentile of their Cumulative Distribution Function (Fig. 2C). This measure can be considered as the equivalent of JND (Just Noticeable Difference) for continuous reports. The mean CRD was 3.97 ± 0.26 morph units for radiologists and 4.08 ± 0.25 morph units for untrained observers.

**Fig. 3:**
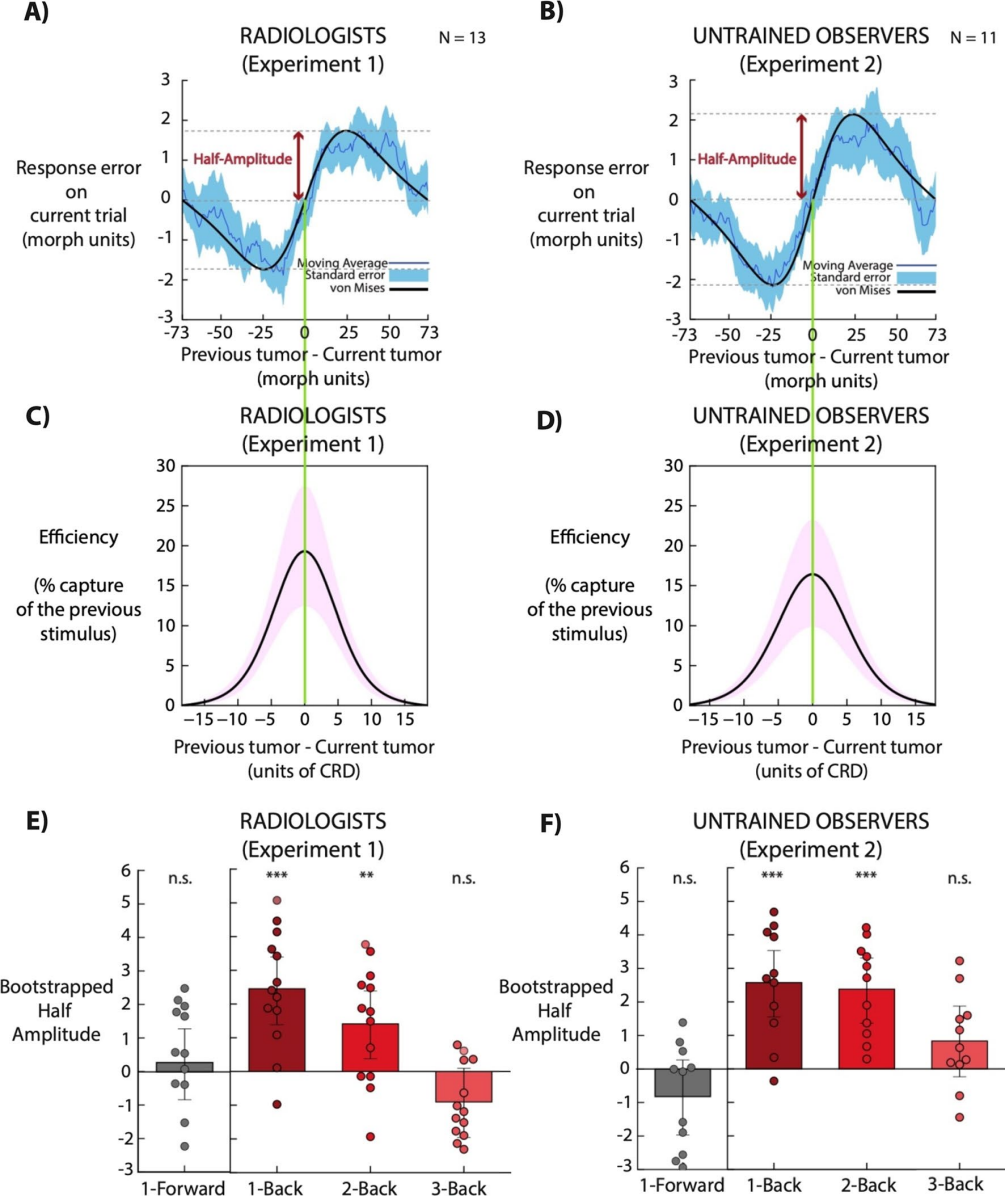
Serial dependence in the perception of simulated lesions by expert radiologists and untrained observers. **A**, **B** In units of shape morph steps, the *x*-axis is the shortest distance along the morph wheel between the current and one-back simulated lesion, and the *y*-axis is the shortest distance along the morph wheel between the selected match shape and current simulated lesion. Positive x axis values indicate that the one-back simulated lesion was clockwise on the shape morph wheel relative to the current simulated lesion, and positive y axis values indicate that the current adjusted shape was also clockwise relative to the current simulated lesion. The average of the running averages across observers (blue line) reveals a clear trend in the data, which followed a derivative-of-von-Mises shape (model fit depicted as black solid line; fit on average of running averages). Light-blue shaded error bars indicate standard error across observers. Lesion perception was attracted toward the morph seen on the previous trial. Importantly, it was tuned for similarity between previous and current morph (feature tuning). **C**, **D** The derivative-of-von Mises was converted into its source von Mises function (*y*-axis), and the relative morph difference was plotted in terms of CRD units (*x*-axis). Violet shaded error bars indicate 95% confidence interval. The curve indicates the proportion of change in response predicted by the change in the sequential stimulus. **E**, **F** Bootstrapped half amplitudes of derivative of von Mises fit for 1, 2, and 3 trials back. Half amplitude for 1-forward is shown as a comparison (grey bars). Each filled dot represents the bootstrapped half amplitude (morph units) for a single observer. Bars indicate the group bootstrap and error bars are bootstrapped 95% confidence intervals

To test whether radiologists’ lesion perception was pulled by lesions in previous mammograms, we plotted the adjustment error on the current trial in relation to the difference in shape between the current and previous trial, computed as the shortest distance along the morph wheel between the previous lesion and the current lesion. A derivative-of-von Mises curve was then fitted to the observers’ data (Fig. 3A, B, see Feature Tuning analysis). We bootstrapped each subject’s data 5000 times and reported the mean bootstrapped half-amplitude as a metric of the sequential dependence (Fig. 3E, F).

In Experiment 1, all participants except for one displayed a positive von Mises half-amplitude, indicating that lesion perception on a given trial was significantly pulled in the direction of the lesion presented in the previous trial (*p* < 0.001, group bootstrap, *n* = 13, Fig. 3E). Even the lesion two trials in the past influenced current judgments (*p* = 0.01, group bootstrap, Fig. 3E). No attraction was found for 3-trials back (*p* = 0.09, group bootstrap, Fig. 3E). A similar pattern of results was found in Experiment 2 with untrained observers. Lesion perception on a given trial was significantly pulled in the direction of lesions presented in the previous trial for 1 and 2 trials back (*n* = 11; 1-Back; *p* < 0.001, 2-Back; *p* < 0.001, group bootstrap, Fig. 3F) but not for 3-back (*n* = 11; *p* = 0.128, group bootstrap, Fig. 3F). There was no statistical difference between radiologists and untrained observers for 1-back and 2-back (Fig. 3; 1-back, *p* = 0.88; 2back, *p* = 0.19), whereas there was a statistical difference for 3-back (*p* = 0.02; but no serial dependence was detected in those conditions).

As a control for possible confounds or artifacts, we checked whether lesion perception could have been biased from lesions one, two, or three trials in the future. As expected, lesion perception was not significantly influenced by future stimuli for radiologists (1-forward, group bootstrap half amplitude: 0.27 morph units, *p* = 0.50; 2-forward, group bootstrap half amplitude: 0.35 morph units, *p* = 0.5, 3-forward group bootstrap half amplitude: 0.5 morph units, *p* = 0.38). The same was true for naïve observers (1-forward, group bootstrap half amplitude: − 0.83 morph units, *p* = 0.16; 2-forward, group bootstrap half amplitude: 0.22 morph units, *p* = 0.72; 3-forward, group-bootstrap half amplitude: 0.23 morph units, *p* = 0.67).

Average response time was similar across Experiments; 3244 ± 845 ms in Experiment 1 and 2980 ± 578 ms in Experiment 2 (*t*(22) = 0.834, *p* = 0.41). Lesion recognition was therefore strongly attracted toward lesions in previous mammograms seen more than 5 s or 10 s ago (Fig. 3E, F). These results suggest a featural tuning (Fig. 3A, B) and temporal tuning of 5–10 s (Fig. 3E, F), in accordance with previous literature (Fischer & Whitney, 2014; Fritsche et al., 2017; Manassi et al., 2018; Moors et al., 2015; Taubert et al., 2016a; Wexler et al., 2015).

In order to further characterize the strength of the serial dependence effect, we computed how much the current simulated lesion was captured by lesions in the previous trial. We converted the derivative-of-von Mises into its source von Mises function. In order to compare our effect with shape discriminability, we divided the relative morph difference (previous tumor – current tumor; x-axis) by the average CRD index (from Fig. 2C). The plots in Fig. 3B, C show the proportion of change in response (efficiency) predicted by the change in the sequential stimulus. Serial dependence captured the current (simulated) tumor with peaks of 22–25%, and expanded over a large discriminability range (from − 10 to + 10 CRD units).

As an additional analysis, we investigated how much adjustment errors were biased more towards the shape category on the previous trial compared to other previous object categories. Shape categories *A*/*B*/*C* were defined as the prototype *A*/*B*/*C* − / + 24 morph units (49 morph units in total). Adjustment responses were coded as indicating category *A*/*B*/*C*. We computed the percentage of mistakes towards the shape category in 1-back trials, and normalized the index by subtracting 33.33% (chance percentage level) from each percentage index (see Fig. 2 in Manassi et al., 2019 for an in-depth explanation of the analysis). Observers misclassified the simulated lesion on a current trial as the lesion in 1-back trials 8% more often than expected by chance.

**Fig. 4:**
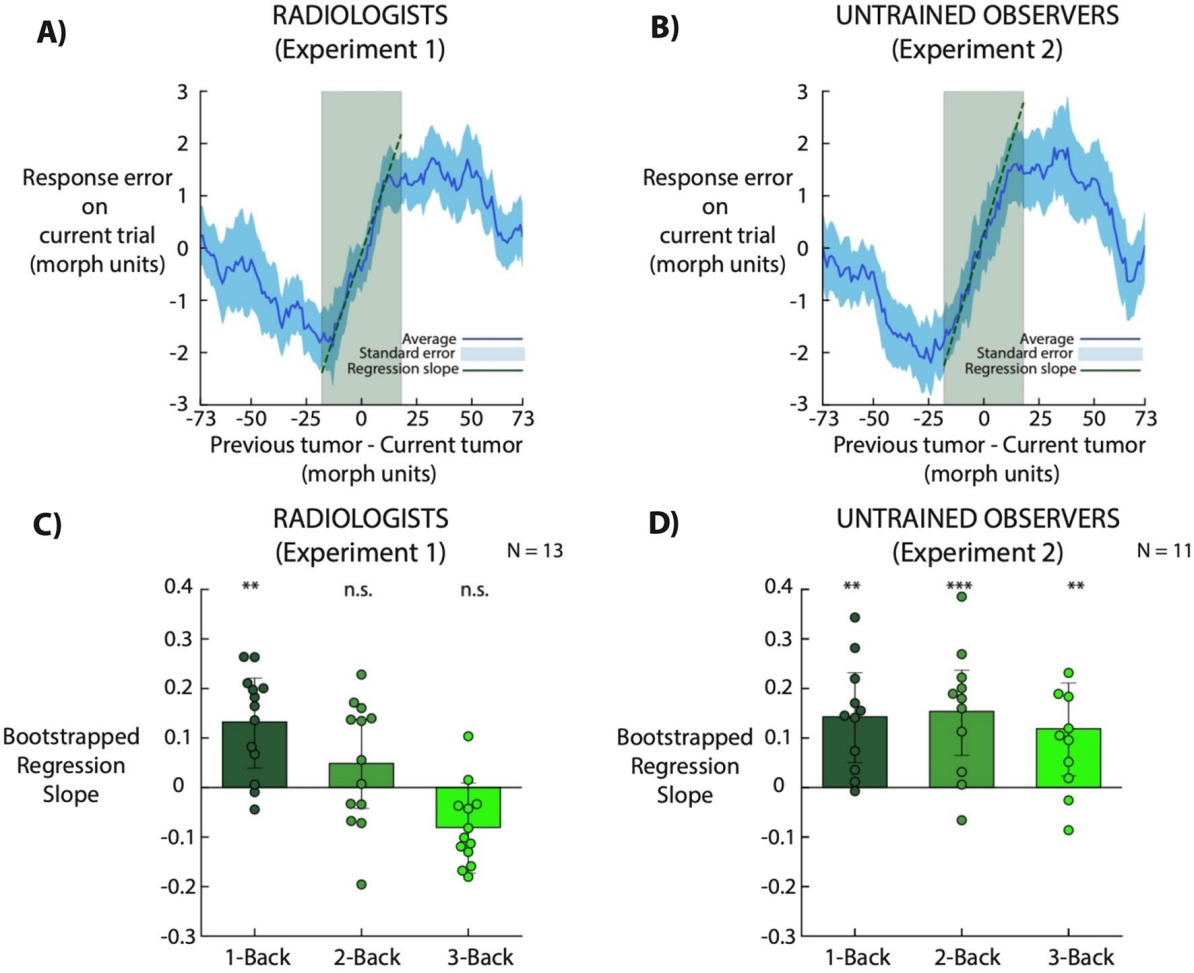
Serial dependence effect size estimation. **A**, **B** Blue lines indicate the average of the running averages across observers (same data as Fig. 2). Light-blue shaded error bars indicate standard error across observers. We fitted a linear regression on the response error as a function of the relative morph difference from − 17 to + 17 morph units (model fit depicted as green dashed line; fit on average of running averages). Dark green shaded areas indicate the morph relative difference considered in the regression analysis. **C**, **D** Bootstrapped regression slopes for 1, 2, and 3 trials back. Each filled dot represents the regression slope for a single observer. Bars indicate the group bootstrap slope and error bars are bootstrapped 95% confidence intervals

In order to further quantify the strength of the 1-back serial dependence effect, we conducted a linear regression analysis on the response error as a function of the relative morph difference (from − 17 to + 17 morph units on the x-axis in Fig. 3A, B, 25% of the central range). Average slope was 0.132 ± 0.10 in Experiment 1 and 0.143 ± 0.10 in Experiment 2, thus meaning that both radiologists and untrained participants exhibited a perceptual pull of ~ 13% towards simulated lesions viewed 1 trial back (Fig. 4, radiologists; 1-back, *p* < 0.01; 2-back, *p* = 0.30; 3-back, *p* = 0.09; naïve observers; 1-back, *p* < 0.01; 2-back, *p* < 0.001; 3-back, *p* = 0.01).

**Fig. 5:**
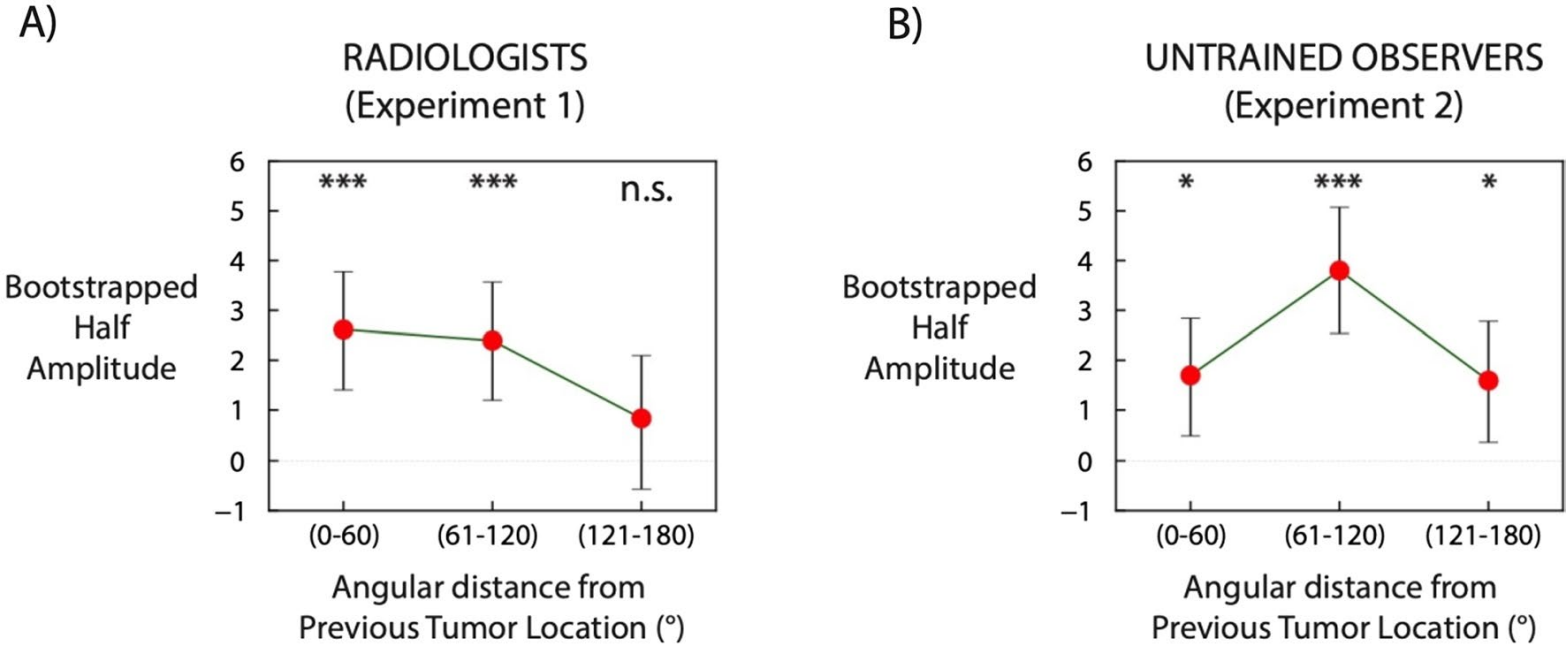
Spatial tuning of serial dependence. **A** refers to Experiment 1, whereas **B** refers to Experiment 2. Each red dot refers to a different relative angular distance between current lesion and lesion in the 1-back trial, super-subject bootstrapped mean. For example, a bin distance 0° indicates that current and previous simulated tumor presented at the same location (30° of angular distance, for example). Error bars are bootstrapped 95% confidence intervals. Dashed line indicates half-amplitude zero (no bias)

As previously mentioned, an important property of serial dependence is spatial tuning (Bliss et al., 2017; Cicchini et al., 2017; Fischer & Whitney, 2014; Fornaciai & Park, 2018; Manassi et al., 2018). We therefore investigated whether serial dependence in simulated radiological screening is affected by the spatial distance between current and previous lesions. On each trial, the simulated lesion was presented at a fixed distance from the center but at random angular distance. Hence, we predicted that serial dependence will be highest when current and previous lesions are presented at a close relative distance, and will gradually decay as relative distance increases. For each participant, we divided the trials into three groups based on the relative distance of the 1-trial back stimulus (Fig. 5; See Spatial Tuning analysis section).

In Experiment 1, serial dependence occurred for an angular distance groups of 0°–60° and 61°–120°, (0°–60°: *p* < 0.001; 61°–120°: *p* < 0.001 group bootstrapped distribution; Fig. 5A), whereas no serial dependence occurred for an angular distance group of 121°–180° (121°–180°: *p* = 0.20; group bootstrapped distribution; Fig. 5A). There was no statistical difference across the two groups for relative distances of 0°–60° (*p* = 0.29), 61°–120° (*p* = 0.11) and 121°–180° (*p* = 0.42). In order to further characterize spatial tuning for 1-trial back, we performed a regression analysis on the three distance groups. Regression slope was significantly different from zero, thus indicating a gradual decay of serial dependence with increased relative distance (slope = − 0.89; *p* = 0.05; group bootstrapped distribution). These results are consistent with prior findings that serial dependence is modulated by the relative location of the sequential targets. Therefore, in a radiological screening environment, the current lesion may be misperceived as more similar to the previous one if current and previous lesions are presented at similar locations. Interestingly, untrained observers from Experiment 2 did not show the same spatial tuning: serial dependence occurred at all tested angular distance groups (0°–60°: *p* < 0.05; 61°–120°: *p* < 0.001; 121°–180°: *p* < 0.05; group bootstrapped distribution; Fig. 5) with no gradual decay as a function of spatial separation. When performing a regression analysis on the three distance groups, regression slope was not significantly different from zero (slope = − 0.05; *p* = 0.90; group bootstrapped distribution; Fig. 5B). The implications of this result will be discussed in the next section.

Taken together, our results show that simulated tumor recognition is strongly biased towards previously presented simulated lesions up to 10 s in the past. Importantly, this sequential effect occurs with expert radiologists and exhibits all the defining properties of traditional serial dependence: feature tuning (Fig. 3A, B), temporal tuning (Fig. 3E, F) and spatial tuning (Fig. 5A).

## Discussion

We found that the perceptual decisions of radiologists were subject to serial dependence. Simulated lesion recognition was biased towards simulated tumors presented up to 10 s in the past (Fig. 3A). Importantly, radiologists exhibited a perceptual pull of ~ 13% towards previously seen tumors (Fig. 4). Moreover, serial dependence alone resulted in 8% more miscategorizations than were expected by chance or due to noise. This perceptual pull exhibited all three tuning characteristics of Continuity Fields: feature tuning (Fig. 3A, B), temporal tuning (Fig. 3E, F) and spatial tuning (Fig. 5A). In Experiment 2, we found largely similar results with untrained observers, with the exception that less clear spatial tuning was found. Taken together, these results show that radiologists’ perceptual judgements are affected by serial dependence.

Our results extend previous work, which investigated the impact of serial dependence in a simulated clinical search task (Manassi et al., 2019). In untrained observers, it was found that shape classification performance was strongly impaired by recent visual experience, biasing classification judgments toward the previous image content. Whereas those results can be considered as a proof of concept that serial dependence can be detrimental in clinical tasks, the present study extended this in several ways including (1) testing trained radiologists, (2) using actual mammogram textured backgrounds as stimuli and (3) implementing a more thorough continuous report task instead of a classification judgment. The results thus show that trained radiologists, as well as naïve observers, suffer from serial dependence. Future research will investigate whether this kind of error occurs in a more realistic radiological screening setting.

Interestingly, we did not find spatial tuning in Experiment 2 with untrained observers. Whereas this seems like a somewhat surprising result, it must be considered that the maximum relative distance in our experiments was 8.8° (double the radius), and previous literature has shown that the spatial window where serial dependence occurs is around 10°–15° or even larger (Collins, 2019; Fischer & Whitney, 2014; Manassi et al., 2019). The potentially interesting result, therefore, is the finding of narrower spatial tuning with expert radiologist observers. The reason for this narrowed spatial tuning is unknown, but it does raise questions about the role of familiarity and expertise. Serial dependence is known to scale with uncertainty (Cicchini et al., 2017), and it is possible that the spatial tuning of serial dependence varies with familiarity as well.

In addition to differences in expertise and familiarity, an additional difference between the two groups of observers in these experiments could be attentional. Previous literature has shown that serial dependence is gated by attention (Fischer & Whitney, 2014; Fornaciai & Park, 2018; Liberman et al., 2016; Rafiei et al., 2021). In comparison to untrained observers, radiologists may pay more attention to the stimuli or attend to different features of the stimuli; therefore, serial dependence tuning may differ with expertise.

It might be argued that our results can be explained by a mere motor response bias, i.e. the motor response during the adjustment task may be biased towards the previous motor response. However, a large literature has shown that serial dependence still occurs when no adjustment is given in the previous trial, thus ruling out a mere motor effect (Fischer & Whitney, 2014; Manassi et al., 2017, 2018). In addition, a simple motor bias cannot explain why serial dependence was tuned for the relative spatial location, biasing simulated tumor judgments only when current and previous tumors were presented at a close angular distance (Fig. 5A). Neither can it explain relative featural difference, biasing tumor adjustment only when current and previous tumors were similar enough (Fig. 3A, B).

Beyond the motor component, there is an intense debate on the underlying mechanism(s) of serial dependence. Among others, serial dependence was proposed to occur on the perception (Cicchini et al., 2017; Fischer & Whitney, 2014; Manassi et al., 2018), decision (Fritsche et al., 2017; Pascucci et al., 2017) and memory level (Barbosa et al., 2020; Bliss et al., 2017). Our results do not allow us to disentangle on which level(s) serial dependence actually occurs. There is psychophysical evidence that serial dependence acts on perception, thus biasing object appearance towards the past (Cicchini et al., 2017; Fischer & Whitney, 2014; Fornaciai & Park, 2019). How serial dependence in perception actually occurs is still a matter of debate; it was recently shown that awareness is required for serial dependence to occur, thus suggesting that a top-down feedback from high level areas is crucial for serial dependence (Fornaciai & Park, 2019; Kim et al., 2020).

It may be argued that the duration of the mammogram presentation (500 ms) is too short and radiologists observe mammograms for a much longer period of time. In fact, the average duration of radiograph fixation for hitting the first mass has been reported as 1.8–2 s, which is surprisingly brief (Krupinski, 1996; Nodine et al., 1996). Interestingly, sufficiently long mammogram exposure durations may lead to the opposite effect, i.e. negative aftereffect. It was found that when adapting normal observers to image samples of dense or fatty tissues, exposure to fatty images caused an intermediate image to appear more dense (and vice versa) (Kompaniez et al., 2013; Kompaniez-Dunigan et al., 2015, 2018). Importantly, mammogram perception was biased away from the past. Future research will establish under which conditions these two biases (perception biased towards or away from the past) arise in radiological screening.

### Limitations of current study

Our results show that radiologists suffer from significant serial dependence in their perceptual judgments. Whether these significant serial dependencies are left at the door of the reading room is as-yet untested. However, the results here show that radiologists are not immune from sequential effects in perceptual decisions. This is only a first step, and there are many improvements required to optimize the ecological validity of our findings. Future improvements will be implemented in order to fully address the impact of serial dependence in a clinical setting.

First, the stimuli. Our study tested serial dependence with a generated set of shape stimuli, but actual tumor images will be required to test the role of serial dependence in radiological screening. In addition, within a radiograph, there can be a variety of features which may be interpreted as tumors, from actual masses, to microcalcifications, architectural distortions, and focal asymmetries. Future research will test whether these features, as well as actual lesions, suffer from serial dependence.

Second, the task. We chose a continuous report paradigm in our experiments, as it provides precise trial-wise errors and has proven to be very reliable in measurements of serial dependence in the past (Cicchini et al., 2017, 2021; Fritsche & de Lange, 2019; Fischer & Whitney, 2014; Fritsche et al., 2017; Liberman et al., 2014). Given the radiologists’ time constraints and resulting limited number of trials, we considered this task to be relatively efficient. The untrained observer data provides a useful baseline in this respect. A previous paper that used a 3AFC classification task found a similar amount of serial dependence in untrained observers as that found here (Manassi et al., 2019). Nevertheless, as the actual task of the radiologist involves classifying lesions and localizing them, implementing more realistic tasks with radiologists will be important in future studies.

Third, mammogram duration. Although radiologists fixate radiographs for slightly longer durations (500 ms in the present and 1.8–2 s reported in the literature; Krupinski, 1996; Nodine et al., 1996), they were shown to perform above chance in detecting abnormalities in chest radiographs with 200 ms duration (Kundel & Nodine, 1975). It will be interesting to test which biases arise with increasing stimulus duration, whether a positive one (as shown by our results), a negative one (Kompaniez et al., 2013; Kompaniez-Dunigan et al., 2015, 2018), or no bias at all.

Finally, whereas our results may indicate that radiological screening is detrimentally affected by serial dependence, they also open avenues to mitigate this bias. Since serial dependence was shown to occur only under restricted featural, spatial, and temporal conditions, some strategies could be implemented to induce perceptual decisions outside of these conditions. For example, mammograms could be presented at different spatial locations. Because of spatial tuning, the relative distance between lesions would be so large that serial dependence would no longer occur. Other strategies may be implemented based on temporal and featural tuning as well.

## Data Availability

All relevant data are available from the authors under request.
